# Treatment of iron deficiency in patients with chronic kidney disease: A prospective observational study of iron isomaltoside (NIMO Scandinavia) 

**DOI:** 10.5414/CN109474

**Published:** 2019-01-07

**Authors:** Gert Jensen, Lasse G. Gøransson, Anders Fernström, Hans Furuland, Jeppe H. Christensen

**Affiliations:** 1 Department of Molecular and Clinical Medicine/Nephrology, The Institute of Medicine, Sahlgrenska Academy, University of Gothenburg, Gothenburg, Sweden,; 2 Department of Internal Medicine, Stavanger University Hospital, Stavanger, Norway,; 3 Department of Nephrology and Department of Medical and Health Sciences, Linköping University, Linköping, Sweden,; 4 Department of Medical Sciences, University Hospital, Uppsala, Sweden, and; 5 Department of Nephrology, Aalborg University Hospital, Aalborg, Denmark

**Keywords:** chronic renal failure, hemoglobin, intravenous iron, iron deficiency anemia, iron isomaltoside

## Abstract

Aims: Iron deficiency is common in patients with chronic kidney disease (CKD). Appropriate iron substitution is critical and intravenous iron is an established therapy for these patients. The objective of this study was to assess treatment routine, effectiveness, and safety of iron isomaltoside (Monofer^®^, Pharmacosmos A/S, Holbaek, Denmark) in CKD patients in clinical practice. Materials and methods: This was a prospective observational study conducted in predialysis CKD patients treated with iron isomaltoside according to the product label and to routine clinical care. Results: The study included 108 patients with predialysis CKD: 22 were in stage 2 – 3, 41 in stage 4, and 45 in stage 5. The mean (standard deviation) age was 67 (15) years, and 55% of patients were male. The majority of patients (65%) received one iron isomaltoside treatment. In patients with a baseline Hb < 10 g/dL, the mean dose of iron isomaltoside in the study was lower than the estimated total iron requirement (567 mg versus 921 mg). A treatment response of Hb ≥ 1 g/dL was achieved in 16/28 (57%) of patients, and the mean post-treatment Hb level was 10.5 g/dL. The probability of retreatment did not correlate with dose, but no dose administered was > 1,000 mg. There were no serious adverse drug reactions. One nonserious adverse drug reaction – injection site discoloration – was reported, and the patient had an uneventful recovery. Conclusion: Iron isomaltoside shows a good effectiveness and safety profile in predialysis CKD patients. However, some patients did not receive adequate iron doses to allow for optimal correction of their iron deficiency anemia.

## Introduction 

Iron deficiency anemia (IDA) is commonly associated with chronic diseases, such as chronic kidney disease (CKD) [1]. Patients with CKD may become iron deficient for various reasons including: blood loss during dialysis, use of erythropoiesis-stimulating agents (ESAs), and chronic inflammation where enhanced hepcidin levels block the intestinal absorption of iron and its release from iron stores [2, 3, 4]. Iron deficiency, combined with inadequate erythropoietin production by the kidneys and erythropoietin hyporesponsiveness, results in compromised erythropoiesis, and anemia [3, 5]. Consequences of anemia include reduced quality of life [6], CKD progression [7, 8], and increased risk of cardiovascular morbidity and mortality [7]. 

Iron supplementation plays a major role in the management of anemia in CKD patients [9]. The current international treatment guidelines recommend the use of iron irrespective of the use of ESA [10, 11]. In predialysis CKD patients, the use of intravenous (IV) iron is recommended as it is more effective than oral iron to replace the iron stores, and the risk of gastrointestinal side-effects is lower [12]. 

Iron isomaltoside (Monofer^®^, Pharmacosmos A/S, Holbaek, Denmark) is an IV iron that can be administered in doses up to 20 mg per kg body weight, and thus provides the opportunity for correction of iron deficits in only one visit [13]. Iron isomaltoside comprises a matrix structure of iron oxyhydroxide with short, linear, unbranched chains of the carbohydrate isomaltoside (average size, 5.2 glucose units) [13]. This tightly-bound complex of iron and isomaltoside enables a slow and controlled release of bioavailable iron to iron-binding proteins, with little risk of toxicity from labile iron [13, 14]. Hence, iron isomaltoside offers the flexibility to increase the dose of iron. In clinical trials, iron isomaltoside has demonstrated a good efficacy and safety profile in patients with CKD [15, 16, 17]. To date, more than 6,000 patients have been treated within clinical studies with iron isomaltoside, and more than 10 million doses have been used in clinical practice (Data on file, Pharmacosmos A/S, Holbaek, Denmark). 

The present study aimed to assess treatment routine, effectiveness, and safety of iron isomaltoside therapy in CKD patients. The primary endpoint was to determine the probability of needing retreatment over time, according to the dose of iron isomaltoside administered during the first treatment. The underlying hypothesis was that the probability of needing retreatment over time would decrease with increasing iron isomaltoside dose, as demonstrated in patients with gastrointestinal disorders [18]. Such a finding would provide further clinical guidance for optimized IV iron use in predialysis CKD patients. 

## Materials and methods 

### Study design and population 

Participants were recruited from six clinical sites across Denmark, Norway, and Sweden, into a prospective, observational, multicenter study that was conducted between August 2013 and November 2015. The study population included patients (aged ≥ 18 years) with CKD stages 1 – 5 in the predialysis phase who were diagnosed with IDA according to local clinical guidelines. Patients were treated with iron isomaltoside as standard treatment, according to the product label and local clinical practice. Patients were followed prospectively until 2 infusions with iron isomaltoside had been administered or were observed for a minimum of 12 months after the first treatment. All patients were treated and followed according to the local guidelines in each center. The dose of iron isomaltoside and frequency for follow-up were determined by the local clinical practice. At each participating center, a senior nephrologist was responsible for the conduction of the study. 

### Data collection and outcome measures 

Data for iron isomaltoside treatment were collected for up to two treatments per patient. The doses of iron isomaltoside administered during the study were recorded and compared with the estimated total iron need calculated according to the Ganzoni formula [19]. The Ganzoni formula was modified for CKD patients by adjusting the target Hb level to 11.5 g/dL (from the usual 15.0 g/dL). Use of concomitant medications (oral iron, ESA, and blood transfusion), and pre- and post-treatment blood test results were recorded. All data were collected from the medical records. All blood tests were analyzed locally at each participating center. Treatment response was defined as an increase in Hb of ≥ 1 g/dL. Anemia was defined using the limits for Hb outlined by the World Health Organization (WHO) [20]; a second definition of anemia, based on Hb < 10 g/dL, was also considered relevant for this study cohort, given that the patients were typically on Hb maintenance therapy aiming to achieve an Hb level of 10 – 12 g/dL. Adverse drug reactions (ADRs) were registered and reported to the Sponsor’s pharmacovigilance department, and in accordance with the national reporting systems. The collected data were systematically entered into an electronic case report form (eClinicalOS, Merge Healthcare, Morrisville, NC, USA; licensed by BioStata ApS, Birkerød, Denmark). 

### Statistical methods 

All data analyses were conducted on the full analysis set (n = 108), which included all patients who were enrolled in the study according to the protocol criteria and who received at least one dose of the study drug. 

Data are presented as mean and standard deviation (SD) and as median and interquartile range (IQR) for continuous variables and number of exposed patients (with proportions) for categorical variables. The probability of needing retreatment with iron isomaltoside over time was analyzed using a Cox proportional hazards model, with dose category and diagnosis group as factors, and baseline Hb as covariate. p-values were obtained from a two-sided Wald test comparing the hazard ratios (HRs) with one. For the pre- to post-treatment changes in blood parameters, mean change estimates were obtained from an analysis of covariance model with dose category and diagnosis group as factors, and pretreatment value as covariate. p-values for blood parameters were obtained from a two-tailed probability of comparing the mean changes to null. The significance cut-off for all analyses was p < 0.05. All data were analyzed using SAS version 9.4 (SAS institute, Cary, NC, USA). 

### Ethical considerations 

The Regional Ethics Committee in Sweden (application number: EPN Lund 2013/231; approval date: April 30, 2013), the Data Protection Official for Research in Norway (application number: 2013/10419; approval date: August 27, 2013), and the Danish Data Protection Agency (application number: 2013-41-1543; approval date: March 8, 2013) approved the study. The study was registered with the ClinicalTrials.gov registry (NCT01900197). All study participants gave written informed consent before inclusion into the study, and the study was performed in accordance with the Declaration of Helsinki and the European Medicines Agency criteria for noninterventional studies [21]. 

## Results 

### Patients 

**Figure 1.**
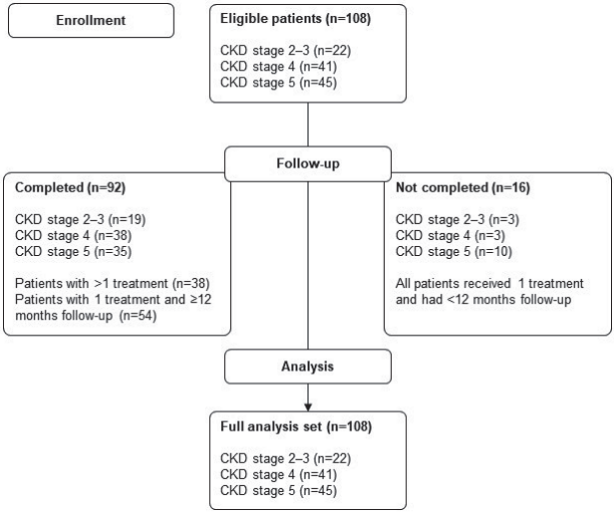
Patient flow diagram. CKD = chronic kidney disease.

The patient flowchart is shown in Figure 1. 108 predialysis CKD patients were included, 22 (20%) patients in CKD stages 2 – 3, 41 (38%) patients in CKD stage 4, and 45 (42%) patients in CKD stage 5. Most patients (n = 92/108; 85%) completed the study, and the majority of these (n = 54/92; 59%) received one infusion with iron isomaltoside and were followed for ≥ 12 months. 

Patient demographics and baseline clinical characteristics are presented in Table 1. The median (IQR) level of C-reactive protein (CRP) was 5 (2 – 13) mg/L for the total population and was highest for patients with CKD stage 5. A total of 30 (28%) patients were anemic, defined as having a level of Hb < 10 g/dL. 

Concomitant medications for treatment of anemia included oral iron (n = 4/108; 4%), blood transfusion (n = 1/108; 1%), ESAs (n = 41/108; 38%), and cobalamin or folic acid (n = 36/108; 33%). ESA use was highest among patients diagnosed with CKD stage 5 (n = 25/45; 56%). Overall, at baseline, the proportion of patients with an Hb level < 10 g/dL was similar between patients receiving ESA (n = 11/41; 27%) and those not receiving ESA (n = 19/67; 28%). 

### Treatment routine and probability of needing retreatment 

The primary endpoint was to determine the probability of needing retreatment over time, according to the dose of iron isomaltoside administered during the first treatment. During a mean (SD) study participation time of 15 (5) months, 70/108 (65%) patients received only one treatment of iron isomaltoside, while 38/108 (35%) patients received two treatments of iron isomaltoside. At the first treatment, the majority of patients (n = 96/108; 89%) were given a dose of < 1,000 mg (mostly 500 mg); the remaining patients received a dose of 1,000 mg. All prescribed doses were administered, in full, in single visits. 

The probability of needing retreatment over time was not significantly different between the patients receiving a dose of < 1,000 mg at the first treatment versus those receiving 1,000 mg (HR: 0.683 (95% confidence interval (CI)): 0.28, 1.64). No patients were treated with doses > 1,000 mg. 

The level of Hb was also evaluated as a predictor for the probability of requiring a second treatment. For each 1 g/dL higher Hb level at baseline (patients matched for diagnosis and treatment dose), there was a trend towards a decreased need of retreatment by 21%, although the finding was not statistically significant (HR: 0.792; 95% CI: 0.60, 1.10). 

### Iron dosing 

Table 2 presents the mean dose of iron isomaltoside administered during the first treatment compared to the mean total iron need calculated using the modified Ganzoni formula with an Hb target of 11.5 g/dL. The actual doses of iron isomaltoside administered were lower than the estimated iron requirement. 

### Effectiveness 

Table 3 presents the mean changes in the levels of Hb, ferritin, and transferrin saturation (TSAT), and the post-treatment levels, measured 6 – 14 weeks following the first iron isomaltoside treatment. Blood parameters showed statistically significant increases for the total patient population. Post-treatment Hb levels reached a mean (SD) of 10.5 (1.4) g/dL for patients with a baseline Hb level of < 10 g/dL. The median (IQR) post-treatment level of ferritin in the total population was 171.0 (120.0 – 238.0) µg/L, and the mean (SD) post-treatment level of TSAT in the total population was 28.4 (9.6)%. 

Following the first iron isomaltoside treatment, 16 of the 28 patients (57%) with an Hb < 10 g/dL at baseline showed a treatment response (increase in Hb of ≥ 1 g/dL). Overall, a post-treatment ferritin level of ≥ 100 µg/L was achieved in 45/54 (83%) patients, and a post-treatment TSAT level of ≥ 20% was achieved in 28/33 (85%) patients, following the first iron isomaltoside treatment. 

The proportion of patients with an Hb level of < 10 g/dL was slightly lower prior to the second iron isomaltoside treatment compared to that observed before the first treatment (24% versus 28%, respectively). 

### Safety 

One nonserious ADR was reported. The patient developed a brown discoloration at the injection site 24 hours after the iron isomaltoside infusion, which may have resulted from a drug administration error causing injection site extravasation. The patient did not experience any swelling, pain, or discomfort and had an uneventful recovery. The event was considered possibly related to the study drug treatment. 

## Discussion 

In this study, treatment of IDA in predialysis CKD with iron isomaltoside showed a good safety profile and resulted in increased levels of Hb, ferritin, and TSAT, despite some patients being underdosed. The underdosing was evident when comparing the iron doses administered in the study (typically < 1,000 mg; mostly 500 mg) with the total iron need calculated using a modified Ganzoni formula with a target Hb of 11.5 g/dL. Post-treatment Hb levels for patients with a baseline Hb < 10 g/dL were, on average, within the target range of 10 – 12 g/dL, albeit at the lower end of the range. This finding indicates that a better treatment effect, e.g., reaching an Hb level of 11.5 g/dL after treatment, could have been achieved with higher iron doses. The probability of a need for retreatment over time did not show any dose-dependency, but this may have been a result of the iron underdosing and/or the fact that no patient received a dose > 1,000 mg. High iron doses, especially > 1,000 mg, have been shown to reduce the need for retreatment in patients with gastrointestinal disorders such as inflammatory bowel disease [18]. Relatively few patients received retreatment during the study, which also resulted in limited power to detect any impact of iron dose. 

Clinical studies calculating the total iron need in predialysis CKD patients using the Ganzoni formula have reported administering single, mean doses of IV iron above 500 mg [15, 17], although 500 mg was the most common dose used in this study. High doses are often required to manage iron deficiency and IDA in various clinical situations, including predialysis CKD, where the administration of IV iron is appropriate [22]. It has been suggested that a total cumulative IV iron dose of > 1,000 mg is representative of the actual iron deficit in predialysis CKD patients with IDA [23]. Indeed, cumulative doses of IV iron in excess of 1,500 mg, over a period of up to 12 months, have been documented in published reports of clinical studies in patients with CKD [5, 24]. 

The underdosing with iron isomaltoside observed in this study may have resulted from targeting an insufficient Hb level for proper anemia correction and administering 500 mg iron doses to patients with advanced IDA (having an Hb < 10 g/dL). For management of anemia using ESA, initiation of ESA treatment in predialysis CKD patients is recommended when the Hb level is < 10 g/dL, and guidelines recommend that ESA is not used to maintain an Hb level > 11.5 g/dL [10]. For Hb levels ≥ 10 g/dL, however, the anemia can be managed with IV iron alone [10] without the risk of achieving supraphysiological levels of Hb given the limited supply of erythropoietin in these patients. It may also be important to treat iron deficiency in nonanemic patients because iron deficiency is, in itself, associated with clinical symptoms. 

In the present study, less than half of the patients received ESA therapy at baseline and, in these individuals, the correction of anemia was not improved compared to those without ESA therapy. This finding suggests that any iron supplementation (oral or IV) administered to patients before entering the study was not sufficient to optimally manage the anemia when combined with ESA. Untreated iron deficiency is an important cause of hyporesponsiveness to ESA therapy, and is one of only a few other easily reversible factors that contribute to a lack of ESA treatment effect on Hb levels [10]. Therefore, it is important to ensure adequate iron stores prior to initiating ESA therapy, as well as during ESA therapy, to maximize the likelihood that the treatment is effective [10]. 

When used appropriately, IV iron can reduce the requirement for ESA therapy and allow a decrease in ESA dose in CKD patients [5, 24], lowering the ESA-associated risk of stroke, thrombosis, serious cardiovascular events, and death [25]. ESA-sparing IV iron therapies may also alleviate the economic burden of anemia management [26]. When considering iron and ESA cotherapy, clinical guidelines primarily recommend IV iron treatment for adults and young people with anemia as a result of CKD who are iron deficient and receiving ESA, and for those who are not receiving hemodialysis, high-dose, low-frequency IV iron is recommended [27]. 

Iron isomaltoside demonstrated a good safety profile, consistent with the findings from clinical trials of iron isomaltoside in CKD patients [15, 16, 28], and a recent meta-analysis that included > 5,000 patients from clinical trials showed a lower frequency of serious and/or severe hypersensitivity reactions with iron isomaltoside compared to iron sucrose and ferric carboxymaltose [28]. There were no hypersensitivity reactions in the current study. A single nonserious event of a brown discoloration at the injection site was reported, and the patient had an uneventful recovery. 

The present study observed routine clinical care in order to inform clinical guidance for the optimization of IV iron treatment. By nature of an observational study, blood testing before and after iron treatment was performed in accordance with local clinical practice and did not always occur at the appropriate time to capture optimal treatment responses; this could have influenced the changes in blood parameter outcomes and the decision for retreatment. Also, no instructions were given on iron dosing, which consisted of mainly 500 mg doses. Combined with limited sample sizes in the dosing groups, there was insufficient power for a robust assessment of dose-dependency on the need for retreatment (primary endpoint). Furthermore, the recurrence of anemia and, subsequently, the need for retreatment can be affected by disease, inflammation, and medications such as angiotensin-converting enzyme inhibitors and acetylsalicylic acid. However, data specific to disease activity, anti-inflammatory treatments, or other therapies that could influence anemia were not recorded during the study. Another limitation of this study is the single-arm design without any control or active comparator group; the primary endpoint of the study was to compare different dose groups of iron isomaltoside treatment. 

In conclusion, this study showed that iron isomaltoside is effective in predialysis CKD and has a good safety profile. However, in this real-life clinical setting, not all patients received adequate iron doses to allow for optimal correction of their IDA. Therefore, focus on the iron dose is needed when managing IDA in these patients. 

## Acknowledgment 

The authors thank all the investigators and study personnel for their contribution to the study as well as BioStata ApS, Birkerød, Denmark, for providing statistical support. From Pharmacosmos A/S, Holbaek, Denmark, a special thank you to Dorte Rytter Nielsen, Malin Winterleijon, and Sylvia Simon for coordinating and supporting the study. The authors also gratefully acknowledge the medical writing assistance provided by Cambridge Medical Communication Ltd., Cambridge, UK. 

## Data availability 

The datasets generated during and/or analyzed during the current study are available from the corresponding author on reasonable request. 

## Funding 

The study centers received a fee per patient for data collection from the Sponsor, Pharmacosmos A/S, Holbaek, Denmark. 

## Conflict of interest 

Gert Jensen, Lasse G. Gøransson, Anders Fernström, Hans Furuland, and Jeppe H. Christensen declare no conflict of interest. 

**Table 1.** Patient demographics and baseline clinical characteristics.

	CKD stage 2 – 3 (n = 22)	CKD stage 4 (n = 41)	CKD stage 5 (n = 45)	Total (n = 108)
Demographic characteristics				
Gender, n (%)				
Female	14 (63.6)	14 (34.1)	21 (46.7)	49 (45.4)
Male	8 (36.4)	27 (65.9)	24 (53.3)	59 (54.6)
Age (years), mean (SD)	58.3 (15.7)	72.8 (11.3)	65.6 (16.3)	66.9 (15.3)
Weight (kg), mean (SD)	85.3 (19.9)	79.9 (15.5)	79.3 (19.0)	80.8 (17.9)
Anemia status				
Anemic patients (WHO criteria)^a^, n (%)	18 (81.8)	40 (97.6)	41 (91.1)	99 (91.7)
Patients with Hb < 10 g/dL, n (%)	4 (18.2)	11 (26.8)	15 (33.3)	30 (27.8)
Clinical characteristics				
Hb (g/dL)	n = 22	n = 41	n = 45	n = 108
Mean (SD)	10.9 (1.5)	10.5 (1.0)	10.5 (1.1)	10.6 (1.2)
Ferritin (µg/L)	n = 21	n = 35	n = 36	n = 92
Mean (SD)	56.2 (62.9)	137.4 (179.1)	132.4 (122.3)	116.9 (140.4)
Median (IQR)^b^	32.0 (14.0 – 66.0)	89.0 (42.0 – 137.0)	98.0 (48.5 – 164.5)	76.5 (34.5 – 130.5)
TSAT (%)	n = 16	n = 29	n = 22	n = 67
Mean (SD)	11.3 (6.8)	18.4 (7.0)	18.5 (8.5)	16.7 (7.9)
CRP (mg/L)	n = 16	n = 35	n = 39	n = 90
Mean (SD)	6.9 (7.7)	10.4 (14.3)	15.9 (24.6)	12.2 (18.9)
Median (IQR)^b^	3.8 (1.9 – 9.6)	3.4 (1.3 – 13.0)	5.3 (2.0 – 21.0)	4.5 (2.0 – 13.0)

^a^Hb < 13 g/dL for men and Hb < 12 g/dL for women. ^b^Data not normally distributed are presented as median values. CKD = chronic kidney disease; CRP = C-reactive protein; Hb = hemoglobin; IQR = interquartile range; n = number of patients; SD = standard deviation; TSAT = transferrin saturation; WHO = World Health Organization.

**Table 2.** Dose of iron isomaltoside administered versus the calculated total iron need for anemic patients receiving the first iron isomaltoside treatment.

	CKD stage 2 – 3	CKD stage 4	CKD stage 5	Total
Anemic patients at baseline (WHO criteria)^a^	n = 15	n = 33	n = 36	n = 84
Dose for the first treatment (mg)	566.7 (175.9)	551.5 (148.2)	555.6 (159.4)	556.0 (156.3)
Total iron need calculated using Ganzoni formula (mg)^b^	795.0 (173.4)	758.0 (142.7)	757.0 (167.1)	764.2 (157.8)
Patients with Hb < 10 g/dL at baseline^c^	n = 4	n = 11	n = 15	n = 30
Dose for the first treatment (mg)	500.0 (0.0)	590.9 (202.3)	566.7 (175.9)	566.7 (172.9)
Total iron need calculated using Ganzoni formula (mg)^b^	1,052.8 (45.4)	913.1 (63.4)	891.3 (168.0)	920.9 (134.5)

Data presented are means (SD) unless otherwise stated. ^a^Hb < 13 g/dL for men and Hb < 12 g/dL for women; patients with an Hb > 11.5 g/dL and patients with missing weight data were excluded to fit the use of the Ganzoni formula. ^b^Ganzoni formula based on the patient’s actual recorded weight, a target Hb of 11.5 g/dL, Hb level prior to the first iron isomaltoside treatment in the study, and an iron store of 500 mg. ^c^Patients with missing weight data were excluded to fit the use of the Ganzoni formula. CKD = chronic kidney disease; Hb = hemoglobin; n = number of patients; SD = standard deviation; WHO = World Health Organization.

**Table 3.** Analysis of blood parameters after the first iron isomaltoside treatment.

	CKD stage 2 – 3	CKD stage 4	CKD stage 5	Total
Hb (g/dL)				
Anemic patients at baseline (WHO criteria)^a^	n = 16	n = 36	n = 38	n = 90
Change from baseline to post-treatment	1.4 (0.8)	0.7 (1.3)	0.8 (1.4)	0.9 (1.3)
p-value^b^	< 0.0001	0.0021	0.0005	< 0.0001
Time of evaluation (weeks)^c^	6.6 (4.1)	9.2 (5.6)	9.7 (11.3)	8.9 (8.4)
Post-treatment level	11.9 (1.1)	11.2 (1.4)	11.1 (1.4)	11.3 (1.4)
Patients with Hb < 10 g/dL at baseline	n = 3	n = 10	n = 15	n = 28
Change from baseline to post-treatment	1.5 (0.7)	1.1 (1.4)	1.3 (1.7)	1.3 (1.5)
p-value^b^	NS	NS	0.0054	0.0001
Time of evaluation (weeks)^c^	7.9 (8.7)	5.9 (3.7)	6.3 (4.2)	6.3 (4.4)
Post-treatment level	10.3 (0.6)	10.4 (1.4)	10.6 (1.6)	10.5 (1.4)
Ferritin (µg/L)				
Change from baseline to post-treatment	n = 8	n = 25	n = 21	n = 54
Mean (SD)	114.0 (150.7)	95.9 (175.9)	111.6 (121.6)	104.7 (150.5)
Median (IQR)^d^	90.0 (18 – 113)	77.0 (46 – 117)	82.0 (43 – 184)	86.0 (38 – 136)
p-value^b^	0.0114	0.0056	0.0011	< 0.0001
Time of evaluation (weeks)^c^	11.6 (18.2)	11.3 (6.4)	10.5 (12.2)	11.0 (10.9)
Post-treatment level				
Mean (SD)	173.4 (213.2)	219.5 (210.1)	242.0 (162.8)	221.4 (191.1)
Median (IQR)^d^	138.0 (37.5 – 173.0)	169.0 (120.0 – 218.0)	200.0 (161.0 – 280.0)	171.0 (120.0 – 238.0)
TSAT (%)	n = 6	n = 17	n = 10	n = 33
Change from baseline to post-treatment	8.7 (7.3)	12.8 (8.7)	10.5 (10.5)	11.4 (8.9)
p-value^b^	0.0242	< 0.0001	0.0148	< 0.0001
Time of evaluation (weeks)^c^	12.8 (21.3)	13.1 (6.5)	13.6 (16.9)	13.2 (13.1)
Post-treatment level	21.7 (5.6)	30.7 (10.1)	28.6 (9.6)	28.4 (9.6)

Data presented are means (SD) unless otherwise stated. ^a^Hb < 13 g/dL for men and Hb < 12 g/dL for women. ^b^p-values reflect the two-tailed probability of comparing the mean change to null. ^c^Number of weeks post-treatment between the last administration of the first iron isomaltoside treatment and the follow-up blood test. ^d^Data not normally distributed are presented as median values. CKD = chronic kidney disease; Hb = hemoglobin; IQR = interquartile range; n = number of patients; NS = not significant; SD = standard deviation; TSAT = transferrin saturation; WHO = World Health Organization.
